# Exploratory genomic stratification of benefit from first-line immunotherapy-based combination in advanced biliary tract cancer: a biomarker analysis of a randomized phase 2 trial

**DOI:** 10.3389/fimmu.2026.1878780

**Published:** 2026-07-27

**Authors:** Yatong He, Zeyu Ruan, Xiaoqing Xu, Yue Han, Junrong Yan, Jiaojiao Ni, Qi Xu, Jieer Ying, Shurui Zhou

**Affiliations:** 1 Postgraduate Training Base Alliance of Wenzhou Medical University (Zhejiang Cancer Hospital), Hangzhou, Zhejiang, China; 2 Department of Hepato-Pancreato-Biliary & Gastric Medical Oncology, Zhejiang Cancer Hospital, Hangzhou Institute of Medicine (HIM), Chinese Academy of Sciences, Hangzhou, China; 3 Department of Oncology, Hangzhou Xixi Hospital, Hangzhou, China; 4 Geneseeq Research Institute, Nanjing Geneseeq Technology Inc., Nanjing, China; 5 Key Laboratory of Prevention, Diagnosis and Therapy of Upper Gastrointestinal Cancer of Zhejiang Province, Hangzhou, China

**Keywords:** biliary tract cancer, DNA damage response, genomic classifier, immunotherapy, tumor mutational burden

## Abstract

**Background:**

Benefit from first-line immunotherapy-based treatment in advanced biliary tract cancer (BTC) is heterogeneous, and selection biomarkers are lacking. We investigated whether genomic features could stratify benefit from intensified immunotherapy-based treatment versus chemotherapy.

**Methods:**

This exploratory biomarker analysis included 58 patients from a randomized phase 2 trial of sintilimab, anlotinib, gemcitabine, and cisplatin (SAGC) versus gemcitabine plus cisplatin (GC) with baseline tumor tissue available for 425-gene targeted next-generation sequencing. Treatment-by-biomarker interactions for progression-free survival (PFS) were evaluated using Cox models. A genomic classifier was developed, and its feature selection robustness and internal stability were assessed by leave-one-out cross-validation (LOOCV) and bootstrap resampling.

**Results:**

Among 58 biomarker-evaluable patients, SAGC improved overall PFS versus GC (median 7.7 vs 6.3 months; HR 0.48, 95% CI 0.27–0.86; P = 0.011), with no overall survival (OS) difference (HR 0.98; P = 0.946). Evaluating these interactions identified DNA damage response (DDR) alteration and high tumor mutation burden (TMB-H) as the most robust predictors. Applied to both endpoints, the classifier stratified patients into SAGC-predominant (SP; either feature, n=40) and GC-predominant (GP; neither feature, n=18) subgroups, revealing diametrically opposed clinical trajectories (interaction P = 0.001 for PFS; P = 0.004 for OS). In SP, SAGC markedly prolonged PFS (10.7 vs 4.5 months; HR 0.26, 95% CI 0.12–0.55; P = 0.0002) and showed a favorable OS trend (16.9 vs 10.3 months; HR 0.55; P = 0.113). Conversely, in GP, SAGC yielded shorter PFS (5.8 vs 8.0 months; HR 2.70; P = 0.078) and significantly shorter OS (8.2 vs 13.9 months; HR 3.73, 95% CI 1.18–11.77; P = 0.017) compared to GC. LOOCV supported the reproducibility of feature prioritization and the internal stability of the final classifier, while bootstrap resampling supported the robustness of subgroup-specific treatment effects.

**Conclusions:**

An exploratory genomic classifier based on DDR alteration and TMB-H may help identify patients with advanced BTC more likely to benefit from the intensified first-line SAGC regimen, pending external validation.

## Introduction

For a long time, first-line treatment for advanced biliary tract cancer (BTC) remained largely limited to gemcitabine plus cisplatin (GC) (1). More recently, the treatment landscape has begun to change, with trials such as TOPAZ-1 and KEYNOTE-966 introducing immunotherapy into the first-line setting (2, 3). This represented an important step forward, but the magnitude of benefit in unselected populations remained modest. In the overall study populations of these trials, the improvement in survival was generally less than two months, suggesting that many patients still do not derive substantial benefit from standard chemo-immunotherapy.

To improve on these results, more intensive combination strategies have been explored. One approach that has attracted increasing interest is the addition of anti-angiogenic agents to chemo-immunotherapy (4–6). The rationale is that vascular targeting may modulate the tumor microenvironment and enhance the antitumor efficacy of immunotherapy through positive feedback loops that reinforce each other (7). We recently evaluated this strategy in our multicenter, randomized phase 2 SAGC trial, which tested the addition of sintilimab and anlotinib to standard gemcitabine plus cisplatin (8). This quadruplet regimen improved tumor response and prolonged median progression-free survival (PFS) to 8.5 months, compared with 6.3 months with chemotherapy alone.

However, despite the clear PFS improvement, no overall survival (OS) benefit was observed. Median OS (mOS) was nearly identical between the two treatment arms (13.2 vs. 13.7 months) (8). This discrepancy is clinically important, because it suggests that the conventional intention-to-treat analysis may be obscuring biologically meaningful heterogeneity within the BTC population. It is plausible that some molecular subgroups derive substantial benefit from the SAGC regimen, whereas others may gain little while being exposed to additional treatment burden.

At present, however, there is no practical way to distinguish these patients. A more practical biomarker-based stratification approach is needed. A similar problem has been addressed in lung cancer research through the MINERVA score, which used a broader genomic signature rather than a single-gene biomarker to identify differential treatment benefit (9). That strategy revealed survival benefits that were not apparent in the overall population. We reasoned that advanced BTC may also benefit from a more precise biomarker-based stratification strategy.

Against this background, we performed large-panel next-generation sequencing (NGS) covering 425 genes in 58 patients from the SAGC trial with sufficient baseline tumor tissue available. The purpose of this analysis was not merely to describe the mutational landscape, but to explore whether genomic features could help explain differential benefit from SAGC versus GC. More specifically, we aimed to identify predictive biomarkers and to explore whether a simple biomarker-based classifier could stratify patients into subgroups with different relative benefit from SAGC versus GC. We also sought to determine whether such stratification could uncover an OS signal in biomarker-enriched subsets, which was not observed in the intention-to-treat population. If successful, this approach could provide a practical step toward more individualized first-line treatment for advanced BTC.

## Methods

### Patient enrollment and data collection

The 58 patients included in this genomic sub-study were drawn from the original 80-patient cohort of our randomized SAGC trial (ClinicalTrials.gov: NCT04300959) (8). Baseline tumor tissue of sufficient quality for 425-gene targeted next-generation sequencing (NGS) was available for 58 patients, including 30 in the SAGC arm and 28 in the GC arm. Of note, 16 biomarker-evaluable patients from the SAGC arm were also longitudinally included in our previously reported prospective observational multi-omics study (NCT06048289) (10). Clinical data, including treatment response and survival outcomes, were obtained from the primary trial database. The trial was conducted in accordance with the Declaration of Helsinki and approved by the Ethics Committee of Zhejiang Cancer Hospital (Approval No.: IRB-2019-203). All patients provided written informed consent.

### DNA extraction, library preparation, and targeted sequencing

Genomic DNA was extracted from archived FFPE tumor tissue using the QIAamp DNA FFPE Tissue Kit (Qiagen). DNA quantity and purity were assessed using a Qubit 3.0 fluorometer and a Nanodrop 2000. Sequencing libraries were prepared with the KAPA Hyper Prep Kit and enriched using the 425-gene GeneseeqPrime^®^ pan-cancer panel with xGen Lockdown reagents. Captured libraries were amplified with KAPA HiFi HotStart ReadyMix (KAPA Biosystems), quantified with the KAPA Library Quantification Kit, and sequenced on Illumina HiSeq 4000 platforms to a mean target coverage depth of at least 1000×.

### Bioinformatics and variant calling

Raw data processing and variant calling followed our previously described pipeline (10) with study-specific refinements. Briefly, Trimmomatic was used for quality control, and clean reads were aligned to the hg19 reference genome using BWA. After duplicate removal and recalibration with Picard and GATK3, somatic single-nucleotide variants (SNVs) and insertion/deletions (indels) were identified using Mutect2. Variants with population frequency >1% in the 1000 Genomes Project or ExAC were excluded. A minimum variant allele frequency threshold of 1% was applied, requiring at least 3 supporting reads for hotspot mutations (≥20 entries in COSMIC v92) and 6 for non-hotspot mutations. All candidate variants were manually reviewed using the Integrative Genomics Viewer (IGV). Gene fusions were identified using FACTERA, and copy number variations (CNVs) were analyzed using ADTEx. Copy number gain and loss were defined by log2 ratio cut-offs of 2.0 and 0.6, respectively.

To characterize pathway-level alterations, curated variants were mapped to the 10 canonical oncogenic signaling pathways and a curated core DNA damage response/repair (DDR) gene set. The DDR panel was compiled from KEGG, Reactome, and Gene Ontology annotations with literature support and was restricted to genes directly involved in canonical DNA repair and checkpoint signaling, including BER, NER, MMR, FA/ICL repair, HR, NHEJ, TLS, and ATM/ATR-mediated checkpoint pathways. Broader DDR-associated genes involved mainly in chromatin remodeling, DNA replication, ubiquitin signaling, or general cell-cycle regulation were excluded. The full gene list is provided in **Supplementary Table 1**.

Tumor mutational burden (TMB) was defined as somatic nonsynonymous mutations per megabase (muts/Mb), including only samples with ≥ 20% ABSOLUTE-estimated tumor purity. Patients were dichotomized into high TMB (TMB-H; ≥5) and low TMB (TMB-L; < 5) subgroups based on the cohort median. Given the typically low mutational burden in advanced BTC, this median cutoff was utilized instead of a universal threshold (e.g., 10 muts/Mb) to ensure balanced group sizes and sufficient statistical power for interaction analyses. Intratumor heterogeneity (ITH; PyClone v0.13.0) and chromosomal instability score (CIS; FACETS) were similarly assessed and dichotomized using the cohort median. Microsatellite instability (MSI) was evaluated using 52 indel loci, and samples with instability in >40% of loci were classified as MSI-high (MSI-H).

### Development of a genomic stratification score to evaluate the relative benefit of SAGC

To identify molecular features associated with differential treatment benefit, treatment-by-biomarker interaction effects were assessed using Cox proportional hazards models. For each candidate biomarker, the interaction effect was evaluated using the following model:


$$h_{i}(t)\;=\;h_{0}(t)\;exp\{\beta_{1}r_{i}\;+\beta_{2}g_{i}\;+\beta_{3}r_{i\;}g_{i}\}$$

where $h_{0}(t)\;$ denotes the baseline hazard, $r_{i\;}$​the treatment assignment for patient i (GC = 0, SAGC = 1), $g_{i}$ ​the biomarker status, $r_{i\;}g_{i}$ the treatment-by-biomarker interaction term. The interaction coefficient $\beta_{3}$ ​was used to estimate the differential treatment effect associated with each biomarker.

To assess whether the interaction effect remained robust after accounting for clinical characteristics, an adjusted Cox model was further fitted:


$$h_{i}(t)\;=\;h_{0}(t)\;exp\{\beta_{1}r_{i}\;+\beta_{2}g_{i}\;+\beta_{3}r_{i\;}g_{i}+\overset{\rho }{\underset{k=1}{\sum }}\gamma k\;X_{ik}\}$$

Where $X_{ik}$ represents the *k*-th clinical covariate for patient i, and γk is the corresponding regression coefficient. The statistical significance of the interaction term $\;\beta_{3}$ in the adjusted model was used to determine whether the biomarker-treatment interaction remained significant after clinical adjustment.

For each biomarker, a standardized interaction statistic ( z-score) was calculated as:


$$z_{g}=\frac{\beta_{3}}{SE(\beta_{3})}$$

where $\beta_{3}$ denotes the treatment-by-biomarker interaction coefficient for biomarker g, and $\;SE\;(\beta_{3})$ its standard error. The sign of the z-score reflected the direction of treatment preference, with negative values indicating relative preference for SAGC and positive values indicating relative preference for GC. Because treatment assignment was randomized, the primary biomarker-selection framework was based on the unadjusted interaction model in order to preserve the randomized comparison structure.

To assess robustness, clinically adjusted interaction analyses were additionally performed using multivariable Cox models including prespecified baseline clinical covariates. Biomarkers showing directionally consistent interaction signals across the unadjusted and adjusted analyses, together with supportive evidence from the adjusted model, were considered for classifier development.

Because the selected biomarkers were binary and showed similar interaction strength in the same SAGC-favorable direction, the final genomic classifier was defined using a simple rule-based framework rather than a continuous weighted score. Patients with neither selected biomarker were assigned to the GC-predominant group (GP), whereas those with either or both biomarkers were assigned to the SAGC-predominant group (SP). Kaplan–Meier analyses were then performed within each subgroup to compare PFS and OS between the SAGC and GC arms.

### Internal validation of the genomic classifier

Internal validation was comprehensively conducted using a two-step leave-one-out cross-validation (LOOCV) approach and bootstrap resampling. First, to address potential overfitting in the biomarker prioritization step, an n−1 LOOCV feature-selection sensitivity analysis was performed. In each of the 58 iterations, one patient was sequentially omitted, and the treatment-by-biomarker interaction analysis was independently re-run on the remaining 57 patients for all 19 candidate genomic features. Features were ranked by interaction P-value in each fold to evaluate whether DDR alteration and TMB-H remained consistently prioritized after removal of individual patients. Second, to evaluate the stability of the final locked classification rule, the omitted patient was reassigned according to the prespecified DDR/TMB-H rule. After completion of all LOOCV iterations, Kaplan–Meier analyses and Cox interaction models were performed using these LOOCV-derived subgroup assignments.

Furthermore, bootstrap resampling was performed for 1, 000 iterations. For each iteration, a bootstrap sample equal in size to the original cohort was generated by sampling with replacement. Cox proportional hazards models were then fitted within each bootstrap sample to estimate treatment hazard ratios in the SP and GP subgroups and the treatment-by-subgroup interaction. Bootstrap estimates were summarized using empirical distributions.

### Statistical analyses

Fisher’s exact test was used to compare categorical variables, and the Wilcoxon rank-sum test was used for continuous variables. PFS and OS were estimated using the Kaplan–Meier method, and differences between groups were assessed with the log-rank test. Hazard ratios (HRs) and corresponding 95% confidence intervals (CIs) were estimated using univariate Cox proportional hazards models. Overlap and association between DDR alteration and TMB-H, the two components of the genomic classifier, were evaluated using contingency counts, overlap proportions, the phi coefficient, and Fisher’s exact test. Collinearity among molecular variables was assessed using variance inflation factors (VIFs). All tests were two-sided, and P < 0.05 was considered statistically significant unless otherwise specified. Statistical analyses were performed using R software (version 4.1.2).

## Results

### Baseline characteristics and clinical outcomes

From our randomized trial, 58 patients with sufficient baseline tumor tissue were included in this genomic sub-study, including 30 in the SAGC arm and 28 in the GC arm. The clinical characteristics of this exploratory cohort were generally well balanced between the two treatment arms (**Table 1**). The median age was 62 years in the SAGC arm and 60 years in the GC arm. Intrahepatic cholangiocarcinoma (ICC) was the most common primary tumor type, accounting for 56.7% of patients in the SAGC arm and 75.0% in the GC arm (P = 0.276). Most patients had metastatic disease, with the liver (70.7%) and distant lymph nodes (65.5%) being the most common sites of involvement. Other baseline characteristics, including sex, Eastern Cooperative Oncology Group (ECOG) performance status, and HBV infection, were comparable between the two groups (all P > 0.05).

**Table 1:** Baseline clinical characteristics of patients enrolled in this study.

Patient chracteristics^a^	SAGC group (n = 30)	GC group (n = 28)	P value
Age (years), median (Range)	62 (34, 73)	60 (32, 78)	0.498
≥ 65	12 (40.0%)	11 (39.3%)	1.000
< 65	18 (60.0%)	17 (60.7%)	
Sex			1.000
Female	10 (33.3%)	10 (35.7%)	
Male	20 (66.7%)	18 (64.3%)	
ECOGb performance status score			1.000
0	1 (3.3%)	0 (0%)	
1	29 (96.7%)	28 (100.0%)	
Primary tumor type			0.276
Extrahepatic cholangiocarcinoma	4 (13.3%)	3 (10.7%)	
Gallbladder carcinoma	9 (30.0%)	4 (14.3%)	
Intrahepatic cholangiocarcinoma	17 (56.7%)	21 (75.0%)	
Metastases			
Liver	20 (66.7%)	21 (75.0%)	0.570
Lung	5 (16.7%)	6 (21.4%)	0.744
Bone	2 (6.7%)	5 (17.9%)	0.246
Peritoneum	7 (23.3%)	5 (17.9%)	0.749
Distant lymph node	20 (66.7%)	18 (64.3%)	1.000

^a^Data are n(%) or medium (Range). ^b^ECOG, Eastern Cooperative Oncology Group.

**Figure 1:**
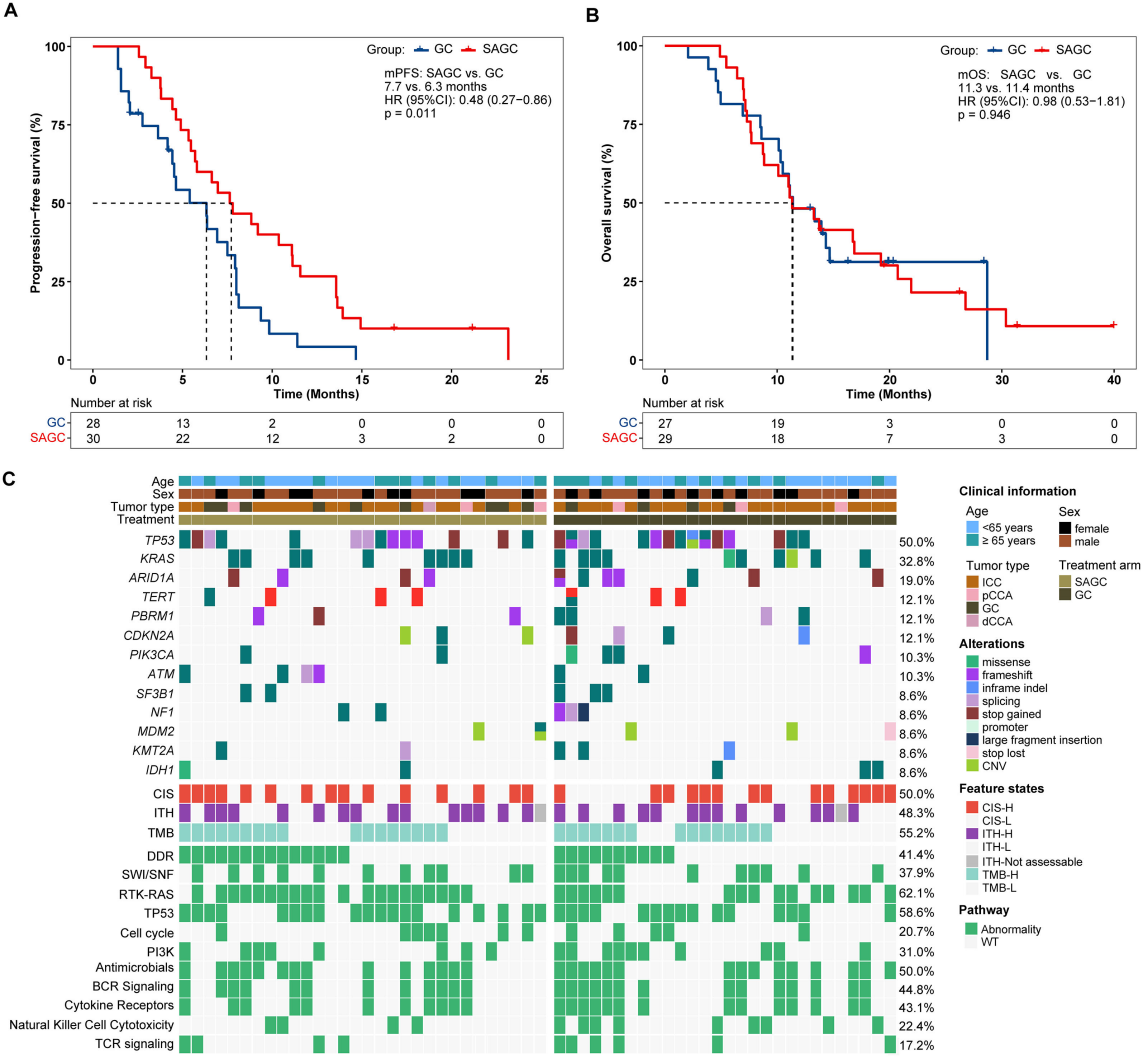
Clinical outcomes and genomic landscape of the biomarker-evaluable cohort. Kaplan–Meier curves for **(A)** progression-free survival (n=58) and **(B)** overall survival (n=56; 2 patients lacked evaluable OS follow-up data) in the SAGC and GC arms. **(C)** Oncoprint of the genomic landscape for 58 patients (30 SAGC, 28 GC). Only features occurring in five patients overall and two patients per arm are shown.

In this biomarker-evaluable cohort, the SAGC arm showed significantly longer progression-free survival than the GC arm. Median PFS (mPFS) was 7.7 months in the SAGC arm versus 6.3 months in the GC arm (HR, 0.48; 95% CI, 0.27–0.86; P = 0.011) (**Figure 1A**). Among the 56 patients with available OS follow-up data, mOS was generally comparable between the two treatment arms (median 11.3 vs. 11.4 months; HR, 0.98; 95% CI, 0.53–1.81; P = 0.946), with no apparent difference observed in this subset (**Figure 1B**). These findings were broadly consistent with the efficacy trend observed in the overall trial population.

### Genomic landscape of the biomarker-evaluable cohort

Comprehensive genomic profiling using the 425-gene panel revealed a mutational landscape consistent with the known biology of advanced BTC. As shown in the oncoprint (**Figure 1C**), only genes, genomic features, and pathway alterations occurring in at least five patients in the overall cohort and in at least two patients in either the SAGC or GC arm are displayed. *TP53* was the most frequently altered gene, occurring in 50.0% of patients, followed by *KRAS* (32.8%) and *ARID1A* (19.0%). Other recurrent alterations included *CDKN2A*, *PBRM1*, and *TERT* (all 12.1%).

At the pathway level, RTK-RAS pathway alterations were the most common, occurring in 62.1% of patients, followed by TP53 pathway alterations (58.6%), antimicrobial response pathway abnormalities (50.0%), B-cell receptor signaling (44.8%), cytokine receptor signaling (43.1%), and DNA damage response pathway alterations (41.4%). SWI/SNF, PI3K, and cell-cycle pathway abnormalities were observed in 37.9%, 31.0%, and 20.7% of patients, respectively, while alterations involving natural killer cell cytotoxicity and T-cell receptor signaling were less frequent (22.4% and 17.2%, respectively). Tumor mutational burden (TMB), chromosomal instability score (CIS), and intratumor heterogeneity (ITH) were ranked across the cohort and dichotomized by the median as high (≥ median) or low (< median), as shown in **Figure 1C**. In addition, two patients were classified as MSI-H, and both belonged to the GC arm; no MSI-H cases were identified in the SAGC arm (data not shown).

### Biomarker-based genomic stratification of treatment benefit

Among all candidates evaluated for treatment-by-biomarker interactions, DDR alteration and TMB-H exhibited the most robust SAGC-favorable signals across both unadjusted and adjusted models (**Supplementary Tables 2**, **3**). To confirm that their selection was not driven by individual outlier patients, we performed an n−1 LOOCV feature selection analysis. Across all 58 folds, DDR alteration and TMB-H were consistently prioritized: DDR ranked 1st in 77.6% of folds (mean rank = 1.28), and TMB-H ranked within the top 3 in 100% of folds (mean rank = 1.86). Notably, both features were simultaneously retained within the top 5 in 100% of folds and within the top 2 in 93.1% of folds (**Supplementary Table 4**), suggesting a clear separation from the remaining 17 candidate features in this exploratory dataset.

Displaying partial overlap and low collinearity (**Supplementary Table 5**), they were combined into a binary classifier: patients with either or both features formed the SAGC-predominant (SP) subgroup (n=40; SAGC: 22, GC: 18), while those with neither formed the GC-predominant (GP) subgroup (n=18; SAGC: 8, GC: 10). The distribution of classifier-defined biomarker subgroups was comparable between the two randomized treatment arms (SP: 73.3% in SAGC vs. 64.3% in GC; Fisher’s exact P = 0.573), supporting baseline balance in this biomarker-evaluable subset. Notably, both MSI-H patients (randomized to GC) fell into the SP subgroup due to concurrent TMB-H. Baseline characteristics were well balanced in the SP subgroup (**Supplementary Table 6**). In the GP subgroup, features were generally comparable except for a baseline imbalance in primary tumor types (P = 0.043); specifically, the SAGC arm contained more poor-prognosis gallbladder carcinomas (37.5% vs. 0.0%), whereas the GC arm was heavily enriched with intrahepatic cholangiocarcinomas (90.0% vs. 37.5%).

**Figure 2:**
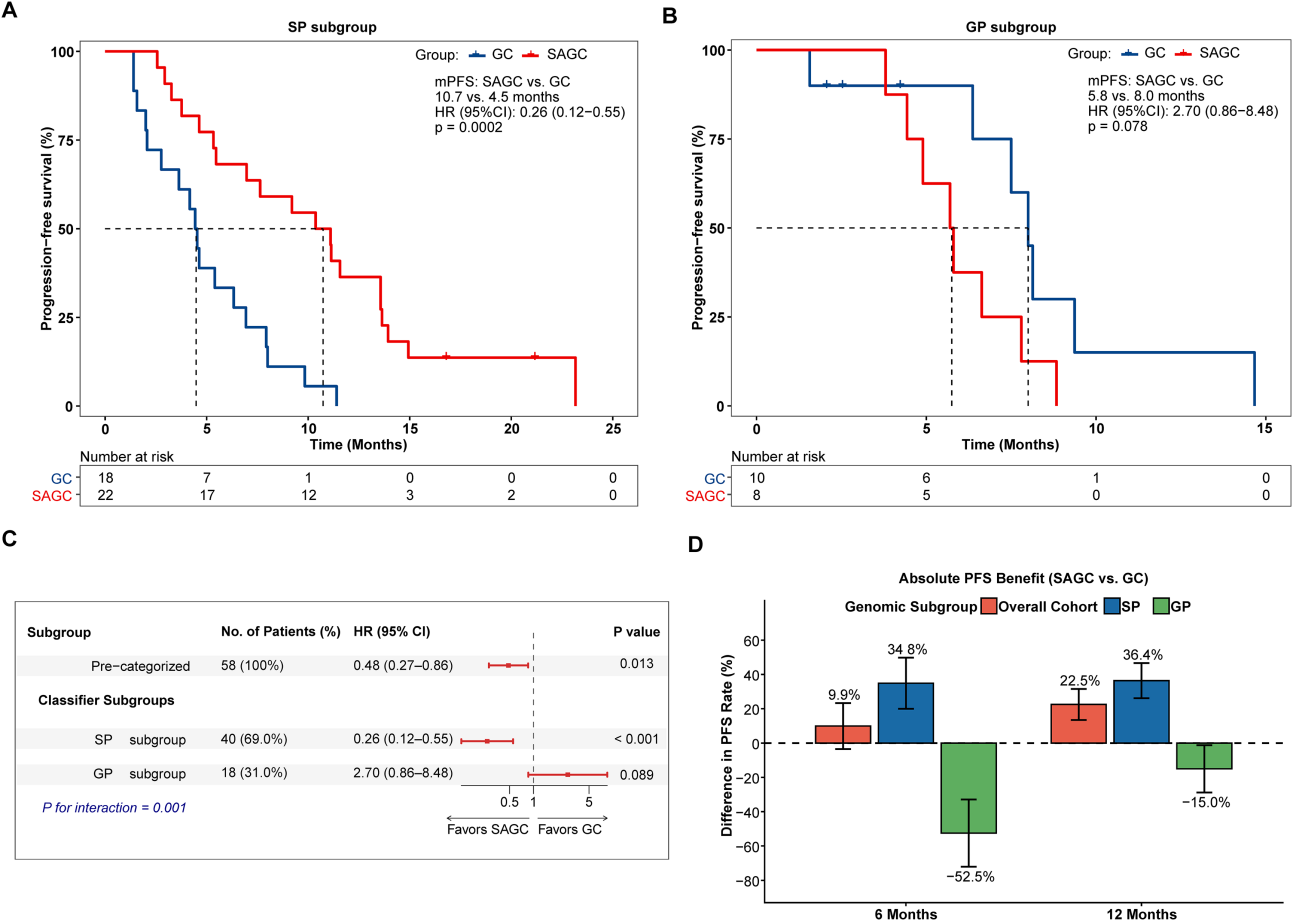
Classifier-based stratification of progression-free survival. Kaplan–Meier curves for progression-free survival by treatment in the **(A)** SAGC-predominant (SP; defined by having DDR alteration, TMB-H, or both) and **(B)** GC-predominant (GP; neither biomarker) subgroups. **(C)** Forest plot comparing relative progression-free survival benefit (hazard ratios) between the overall unselected cohort and the SP/GP subgroups. **(D)** Absolute differences in 6- and 12-month progression-free survival rates (SAGC minus GC). Positive values indicate an absolute benefit from SAGC; negative values indicate a benefit from GC. Error bars represent standard errors.

Classifier stratification revealed strikingly distinct clinical trajectories (P for interaction = 0.001). The average PFS benefit of the unselected cohort (HR 0.48; P = 0.011) was effectively decomposed into a marked SAGC advantage in the SP subgroup (median 10.7 vs. 4.5 months; HR 0.26) and a numerical detriment in the GP subgroup (5.8 vs. 8.0 months; HR 2.70) (**Figures 2A–C**).

Translating these relative risks into absolute metrics (**Figure 2D**) further highlighted this heterogeneity. While the overall unselected cohort showed modest 6- and 12-month absolute PFS gains (9.9% and 22.5%), SAGC yielded substantial absolute benefits of 34.8% and 36.4% in the SP subgroup. Conversely, the intensified regimen was associated with substantial absolute PFS detriments (-52.5% and -15.0%) at corresponding time points in the GP subgroup, highlighting clinically meaningful divergence that was masked in the overall cohort.

### Stratification of OS benefit by the genomic classifier

**Figure 3:**
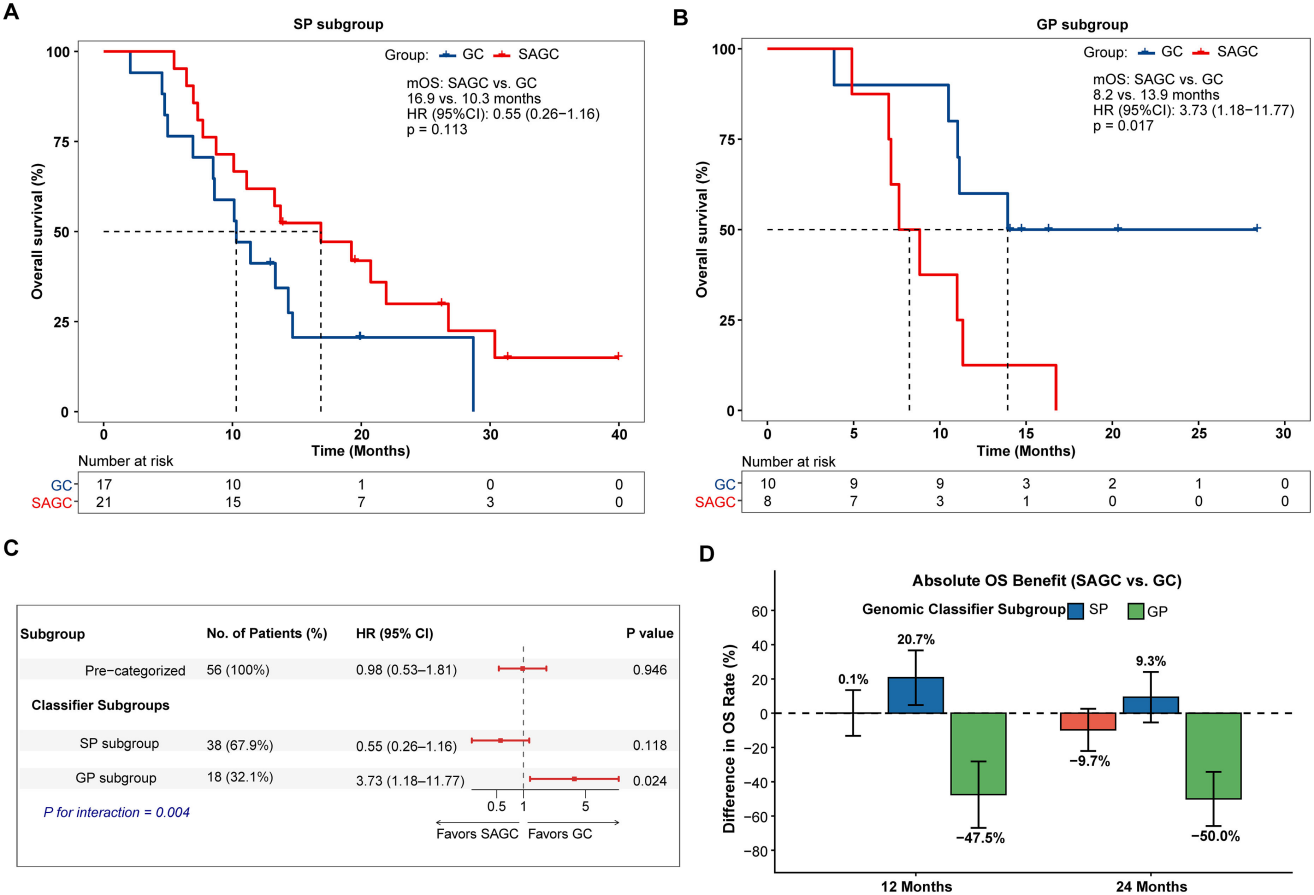
Classifier-based stratification of overall survival. Kaplan–Meier curves for overall survival by treatment in the **(A)** SP and **(B)** GP subgroups. **(C)** Forest plot comparing relative overall survival benefit between the overall unselected cohort and the SP/GP subgroups. **(D)** Absolute differences in 12- and 24-month overall survival rates (SAGC minus GC). Error bars represent standard errors.

Although no meaningful OS difference was observed in the unselected cohort (**Figure 1B**), we investigated whether the PFS-derived classifier could also stratify long-term survival. Consistent with the PFS findings, classifier-based stratification revealed distinctly divergent OS patterns. SAGC yielded a numerically longer median OS in the SP subgroup (16.9 vs. 10.3 months; HR, 0.55; 95% CI, 0.26–1.16; P = 0.113) (**Figure 3A**), whereas GC achieved significantly longer OS in the GP subgroup (13.9 vs. 8.2 months; HR, 3.73; 95% CI, 1.18–11.77; P = 0.017) (**Figure 3B**). As illustrated in **Figure 3C**, the classifier effectively split the null overall effect into two contrasting relative risks (SP HR: 0.55; GP HR: 3.73), confirmed by a highly significant interaction (P for interaction = 0.004).

Similarly, translating these relative risks into absolute survival metrics highlighted marked clinical differences (**Figure 3D**). In the overall unselected cohort, the intensified regimen provided negligible long-term survival gains (0.1% at 12 months) and even an absolute detriment at 24 months (-9.7%). However, within the SP subgroup, SAGC yielded clear absolute OS benefits of 20.7% and 9.3% at the corresponding time points. In stark contrast, the GP subgroup experienced severe survival detriments from SAGC at both 12 (-47.5%) and 24 months (-50.0%). Thus, the classifier successfully identified subgroups with entirely divergent survival trajectories that were otherwise obscured by the averaged treatment effect in the intention-to-treat population. To address potential confounding from the aforementioned anatomical imbalance in the GP subgroup, we performed a multivariable Cox regression incorporating primary tumor type (gallbladder versus non-gallbladder) as a covariate. Retaining all 18 patients, the multivariable model demonstrated that the treatment-by-subgroup interaction remained significant after adjustment for primary tumor type (adjusted interaction P = 0.001 for PFS; P = 0.005 for OS). Within the GP subgroup, after adjusting for tumor type, the detrimental effect of SAGC remained directionally consistent for PFS (adjusted HR 2.26; 95% CI, 0.65–7.89; P = 0.202) and OS (adjusted HR 3.27; 95% CI, 0.92–11.70; P = 0.068) (**Supplementary Table 7**). These findings suggest that while the anatomical imbalance partially contributed to the initial unadjusted effect size, the distinct clinical divergence remained supported by the genomic treatment-by-biomarker interaction.

### Predictive versus prognostic value of the genomic classifier

To rigorously determine whether the classifier was genuinely predictive of treatment benefit rather than merely prognostic of the natural disease course, we compared survival outcomes between biomarker-positive patients (DDR alteration and/or TMB-H; corresponding to the SP subgroup) and biomarker-negative patients (neither biomarker; corresponding to the GP subgroup) within each treatment arm and in the overall cohort (**Supplementary Figure 1**).

Within the SAGC arm, patients in the SP subgroup exhibited dramatically longer survival than those in the GP subgroup for both PFS (median 10.7 vs. 5.8 months; HR 0.31, 95% CI 0.11–0.81; P = 0.012) and OS (median 16.9 vs. 8.2 months; HR 0.28, 95% CI 0.11–0.74; P = 0.006). Strikingly, within the GC arm, this trend was completely reversed. Without the addition of immunotherapy and anti-angiogenic agents, the SP subgroup experienced significantly shorter PFS (median 4.5 vs. 8.0 months; HR 2.48, 95% CI 0.97–6.31; P = 0.049) and a numerically worse OS trend (median 10.3 vs. 13.9 months; HR 2.13, 95% CI 0.75–5.99; P = 0.145) compared to the GP subgroup. Furthermore, when evaluating the overall cohort regardless of treatment assignment, no survival differences were observed between the SP and GP subgroups for either PFS (median 6.6 vs. 6.6 months; P = 0.462) or OS (median 13.3 vs. 11.1 months; P = 0.689). This distinct cross-over interaction suggests that the genomic classifier lacks inherent prognostic value for advanced BTC. Rather, the biomarker-positive panel was associated with favorable outcomes under the immunotherapy-containing SAGC regimen, whereas the opposite pattern was observed under GC alone, supporting its role as a predictive marker of benefit from the intensified regimen.

### Internal validation of the genomic classifier by LOOCV and bootstrap resampling

**Figure 4:**
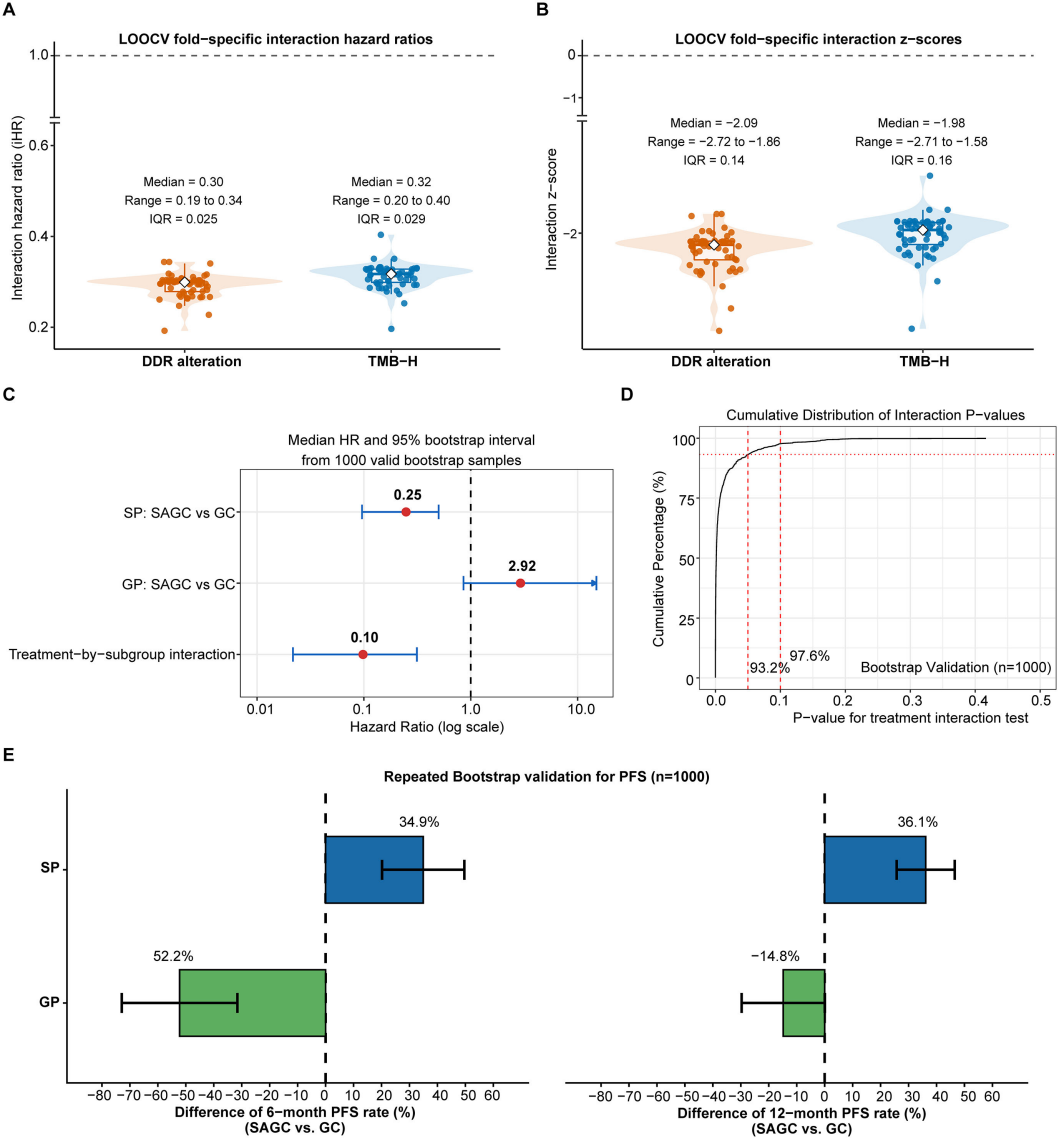
Internal validation of the genomic classifier for progression-free survival. Fold-specific interaction **(A)** z-scores (dashed line: z = 0) and **(B)** hazard ratios (iHRs; dashed line: iHR = 1) across 58 LOOCV iterations. Across 1, 000 bootstrap samples: **(C)** Median bootstrap hazard ratios and treatment-by-subgroup interaction (error bars: 95% intervals; arrows denote truncation). **(D)** Cumulative distribution of interaction P values (dashed lines: P = 0.05 and 0.10). **(E)** Mean absolute differences in 6- and 12-month progression-free survival rates (error bars: standard errors).

Internal validation via LOOCV demonstrated 100% subgroup assignment agreement and a consistently significant treatment-by-group interaction (original iHR = 0.11, P = 0.001). Across all iterations, interaction z-scores and HRs indicated stable SAGC-favorable directionality (**Figures 4A, B**; **Supplementary Table 8**).

Bootstrap resampling (n=1, 000) was then utilized to further confirm this robustness. Across iterations, the interaction remained highly stable (median iHR = 0.10; P < 0.05 in 93.2% of samples). The SP subgroup consistently derived a PFS benefit from SAGC (median HR = 0.25; 100% of samples HR < 1), whereas the GP subgroup repeatedly favored GC (median HR = 2.92) (**Figures 4C, D**; **Supplementary Table 9**). Importantly, these median bootstrap distributions closely mirrored the point estimates derived from the original un-resampled cohort (interaction HR = 0.11; SP HR = 0.26; GP HR = 2.70) (**Supplementary Figure 2A**), demonstrating that the initial findings were computationally stable within this dataset. To translate these relative benefits into clinically intuitive metrics, we evaluated absolute PFS rate differences (**Figure 4E**). In the SP subgroup, SAGC yielded mean absolute increases of 34.9% and 36.1% at 6 and 12 months, respectively. Conversely, the intensified regimen was detrimental in the GP subgroup, causing substantial absolute reductions of 52.2% and 14.8% at corresponding time points.

**Figure 5:**
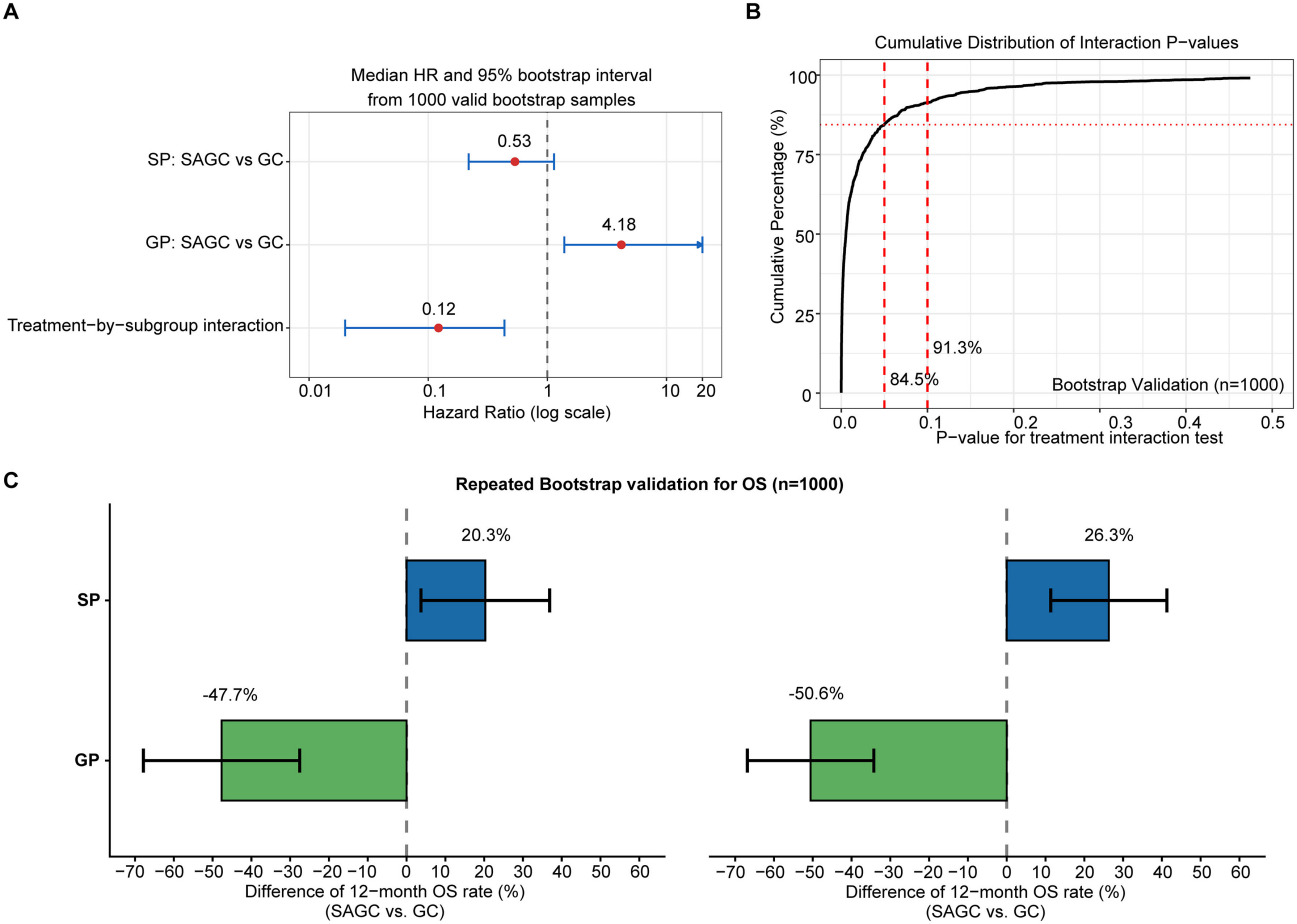
Internal bootstrap validation of overall survival stratification using the locked, progression-free survival-derived classifier. Across 1, 000 bootstrap samples: **(A)** Median bootstrap hazard ratios and treatment-by-subgroup interaction for overall survival (error bars: 95% intervals; arrows denote truncation). **(B)** Cumulative distribution of overall survival interaction P values (dashed lines: P = 0.05 and 0.10). **(C)** Mean absolute differences in 12- and 24-month overall survival rates (error bars: standard errors).

Applying this locked, PFS-derived classifier to 1, 000 bootstrap cohorts successfully validated its utility for OS stratification. The diametrically opposed OS patterns were highly reproducible (**Figure 5A**), supported by a significant treatment-by-subgroup interaction (P < 0.05) in 84.5% of iterations (91.3% for P < 0.10) (**Figure 5B**). Absolute survival metrics mirrored this substantial clinical divergence (**Figure 5C**): SAGC provided mean absolute OS increases of 20.3% and 26.3% at 12 and 24 months in the SP subgroup, while causing severe survival detriments (-47.7% and -50.6%) in the GP subgroup. Similar to PFS, these bootstrap distributions closely mirrored the point estimates from the original un-resampled OS cohort (**Supplementary Figure 2B**), demonstrating that the observed OS divergence was highly reproducible within this specific dataset. Ultimately, these resampling results confirm the classifier’s internal mathematical stability, supporting the credibility of the observed heterogeneity within this exploratory cohort.

## Discussion

Although immunotherapy-based combinations have reshaped the first-line treatment landscape of advanced BTC, its clinical benefit in unselected populations remains modest, and many patients still fail to derive meaningful long-term benefit. This underscores the need for biomarkers that can move treatment decisions beyond a uniform treatment strategy. Our previous SAGC trial (8) illustrated this challenge clearly: despite a significant improvement in PFS with sintilimab, anlotinib, gemcitabine, and cisplatin, no OS benefit was observed in the intention-to-treat population. This discrepancy suggested that treatment sensitivity in advanced BTC is biologically heterogeneous and that the benefit of such intensified regimens may be confined to molecularly defined subgroups.

In the present study, we used large-panel NGS data from the biomarker-evaluable subset of the SAGC trial to explore genomic determinants of relative treatment benefit and to develop an exploratory treatment-selection classifier. Rather than focusing on prognosis alone, our analysis specifically assessed treatment-by-biomarker interactions and identified two features—DDR alteration and TMB-H—that consistently showed SAGC-favorable signals. Based on these findings, we defined a simple classifier that separated patients into a SP group and a GP group.

Several observations deserve emphasis. First, the genomic classifier helped translate divergent relative risks into clinically intuitive absolute survival differences. In this exploratory cohort, patients in the SP subgroup derived notable absolute survival gains from the SAGC regimen, including an observed >34% increase in the 6-month PFS rate and an approximately 20% increase in the 12-month OS rate. Conversely—and importantly for future exploratory clinical consideration—the classifier identified a GP subgroup where the intensified regimen did not provide additional benefit and might even pose an unnecessary treatment burden. For these patients, standard GC alone was associated with better long-term survival, suggesting that such stratification could eventually help spare patients from the toxicities and financial burden of unnecessary immune and anti-angiogenic agents. This divergent benefit pattern was supported by significant treatment-by-subgroup interactions for both PFS and OS. Importantly, our intra-arm survival analyses confirmed a striking cross-over interaction—where the SP subgroup showed superior outcomes under SAGC but worse outcomes under GC—suggesting that the classifier is not merely prognostic of the natural disease course, but genuinely predictive of treatment benefit from the intensified regimen. Thus, the biomarker-positive panel appears to identify patients more likely to derive favorable outcomes from the immunotherapy-containing SAGC regimen, rather than from GC alone. Furthermore, our internal validation using LOOCV and 1, 000-iteration bootstrap resampling indicated that these mathematical trajectories were internally stable. Together, these findings imply that the lack of an OS difference in the overall trial population may reflect the averaging of differential treatment effects across biologically distinct subgroups, rather than a uniformly modest benefit for all patients. However, given the limited sample size (n=18) and the retrospective nature of this subset, the survival detriment observed in the GP subgroup should be interpreted with caution as a hypothesis-generating signal rather than a definitive conclusion.

The biological basis of these findings is plausible. DDR alterations may increase genomic instability and neoantigen generation, thereby enhancing tumor immunogenicity and potentially improving sensitivity to PD-1 blockade therapies (11–15). Likewise, TMB-H has been associated, although not consistently, with greater responsiveness to immunotherapy across tumor types (16–21). In our dataset, these two biomarkers showed only partial overlap and low collinearity, suggesting that they capture related but not redundant aspects of tumor biology. Their combination therefore appears to provide a more informative treatment-selection signal than either feature alone. Notably, the only two MSI-H patients in our cohort (both randomized to the GC arm) were assigned to the SP subgroup due to their concurrent TMB-H status. Although MSI-H is a well-established predictor of immunotherapy benefit, these two patients received standard chemotherapy alone and exhibited mixed clinical outcomes (one progressed rapidly at 2.0 months, while the other reached 9.8 months). Consequently, their presence did not artificially inflate the efficacy of the GC arm within the SP subgroup. This further substantiates that the favorable survival pattern observed in the SP subgroup was genuinely driven by the intensified SAGC regimen, rather than being confounded by the inherently favorable biology of MSI-H tumors.

To contextualize our findings, we must consider the landmark TOPAZ-1 and KEYNOTE-966 trials, which established standard chemo-immunotherapy in advanced BTC. Based on publicly available exploratory analyses from TOPAZ-1, including mutation-status and long-term survivor analyses (22, 23), features such as BRCA mutations appear to define molecular subsets with favorable signal, although these data remain insufficient to establish definitive predictive value for the added efficacy of PD-(L)1 blockade. Crucially, unlike standard dual-chemotherapy plus PD-(L)1 blockade, the SAGC regimen incorporates the anti-angiogenic agent anlotinib. We hypothesize that anlotinib-mediated vascular normalization alleviates local immunosuppression, synergizing with PD-1 blockade to amplify immune responses and “unlock” the specific predictive potential of TMB-H and DDR-induced neoantigens. Consequently, the observed survival advantage in the SP subgroup cannot be solely attributed to sintilimab. Rather, our genomic classifier should be interpreted as a regimen-specific composite predictor for the overall intensified SAGC regimen.

We recently reported a multi-omics analysis from a prospective observational registry (NCT06048289) (10), which shares a minor overlap (n=16) with our investigational arm. However, the current sub-study of a distinct randomized trial (NCT04300959) addresses a fundamentally different clinical question. While the prior observational study identified prognostic features for standard chemo-immunotherapy using complex RNA profiles, it inherently could not isolate predictive utility. By leveraging a randomized design and formal interaction analysis, the present study provides stronger evidence to distinguish predictive biomarkers from pure prognostic effects. Furthermore, we evaluate an intensified quadruplet regimen incorporating anti-angiogenesis, delivering a pragmatic, DNA-only classifier (DDR alteration and TMB-H) tailored for routine clinical decision-making.

Several limitations should be acknowledged. First, this was a retrospective exploratory analysis. Because biomarker selection and classifier evaluation were performed on the same small dataset (n=58), there is an inherent risk of overfitting. Although our n−1 LOOCV analysis robustly confirmed that the prioritization of DDR alterations and TMB-H was reproducible and not driven by individual outlier patients, internal validation cannot fully eliminate broader overfitting risks, making external validation absolutely necessary. Second, biomarker screening was primarily based on PFS, rendering the OS stratification supportive rather than definitive. Third, simplifying the classifier into a binary rule may reduce biological granularity. Finally, findings in the GP subgroup (n=18) must be viewed strictly as hypothesis-generating. This is compounded by a baseline enrichment of poor-prognosis gallbladder carcinomas in the SAGC arm. Although our multivariable adjustment confirmed that the treatment-by-biomarker interaction survives correction for tumor type, the adjusted estimates in such a limited sample size remain statistically fragile and warrant highly cautious interpretation.

## Conclusions

In summary, our study suggests that a simple genomic classifier based on DDR alteration and TMB-H may help identify patients with advanced BTC who are more likely to benefit from SAGC rather than GC. More broadly, these findings support the value of biomarker-guided treatment selection in uncovering clinically meaningful heterogeneity that may remain hidden in unselected populations. If validated externally, this classifier may have potential clinical utility in guiding first-line treatment selection for advanced BTC.

## Data Availability

The original contributions presented in the study are included in the article/**Supplementary Material**. Further inquiries can be directed to the corresponding authors.

## Supplementary material

Supplementary Figure 1 Kaplan–Meier estimates of progression-free survival and overall survival comparing biomarker-positive versus biomarker-negative patients within the SAGC arm **(A, B)**, the GC arm **(C, D)**, and the overall cohort **(E, F)**. Biomarker-positive denotes patients with DDR alteration and/or TMB-H; biomarker-negative denotes patients with neither biomarker.

Supplementary Figure 2 Original Cox model estimates of progression-free survival and overall survival in the un-resampled cohort.
