# Supplementary material for: Exploratory genomic stratification of benefit from first-line immunotherapy-based combination in advanced biliary tract cancer: a biomarker analysis of a randomized phase 2 trial
Source: Front Immunol. 2026 Jul 27;17:1878780. doi: 10.3389/fimmu.2026.1878780 (PMC13454070; doi:10.3389/fimmu.2026.1878780)
Supplement: Supplementary file 1 [file Table1.docx]

Table S1. Genes included in the DNA damage response pathway panel used for DDR alteration assessment.

| HR | NER | Damage sensing and signaling | BER | MMR | FA | NHEJ | TLS | DR |
| --- | --- | --- | --- | --- | --- | --- | --- | --- |
| MRE11A | CUL5 | ATM | PARP1 | EXO1 | FANCA | LIG4 | POLN | ALKBH2 |
| NBN | ERCC1 | ATR | POLB | MLH1 | FANCB | NHEJ1 | POLQ | ALKBH3 |
| RAD50 | ERCC2 | ATRIP | APEX1 | MLH3 | FANCC | POLL | REV1 | MGMT |
| TP53BP1 | ERCC4 | CHEK1 | APEX2 | MSH2 | FANCD2 | POLM | REV3L |  |
| XRCC2 | ERCC5 | CHEK2 | FEN1 | MSH3 | FANCI | PRKDC | SHPRH |  |
| XRCC3 | ERCC6 | MDC1 | TDG | MSH6 | FANCL | XRCC4 |  |  |
| BARD1 | POLE | RNMT | TDP1 | PMS1 | FANCM | XRCC5 |  |  |
| BLM | POLE3 | TOPBP1 | UNG | PMS2 | UBE2T | XRCC6 |  |  |
| BRCA1 | XPA | TREX1 |  |  |  |  |  |  |
| BRCA2 | XPC |  |  |  |  |  |  |  |
| BRIP1 |  |  |  |  |  |  |  |  |
| EME1 |  |  |  |  |  |  |  |  |
| GEN1 |  |  |  |  |  |  |  |  |
| MUS81 |  |  |  |  |  |  |  |  |
| PALB2 |  |  |  |  |  |  |  |  |
| RAD51 |  |  |  |  |  |  |  |  |
| RAD52 |  |  |  |  |  |  |  |  |
| RBBP8 |  |  |  |  |  |  |  |  |
| SHFM1 |  |  |  |  |  |  |  |  |
| SLX1A |  |  |  |  |  |  |  |  |
| TOP3A |  |  |  |  |  |  |  |  |

BER, base excision repair; NER, nucleotide excision repair; MMR, mismatch repair; FA, Fanconi anemia pathway; HR, homologous recombination; NHEJ, non-homologous end joining; DR, direct repair; TLS, translesion synthesis.
