# Supplementary material for: Exploratory genomic stratification of benefit from first-line immunotherapy-based combination in advanced biliary tract cancer: a biomarker analysis of a randomized phase 2 trial
Source: Front Immunol. 2026 Jul 27;17:1878780. doi: 10.3389/fimmu.2026.1878780 (PMC13454070; doi:10.3389/fimmu.2026.1878780)
Supplement: Supplementary file 2 [file Table2.docx]

Table S2. Predictive values of genomic aberrations for progression-free survival (unadjusted).

| Genomic aberrations subgroup | iHR (95%CI) | z-score | P value |
| --- | --- | --- | --- |
| *TP53* mutation | 0.395 (0.128-1.214) | -1.621 | 0.105 |
| *KRAS* mutation | 2.458 (0.677-8.930) | 1.366 | 0.172 |
| *ARID1A* wildtype | 1.936 (0.476-7.874) | 0.923 | 0.356 |
| *PBRM1* muation | 0.673 (0.133-3.407) | -0.479 | 0.632 |
| *TERT* mutation | 0.218 (0.032-1.473) | -1.562 | 0.118 |
| TMB-H | 0.312 (0.101-0.961) | -2.030 | 0.042 |
| CIS-H | 2.278 (0.753-6.896) | 1.457 | 0.145 |
| ITH-H | 0.919 (0.303-2.792) | -0.149 | 0.882 |
| DDR pathway abnormality | 0.291 (0.094-0.899) | -2.145 | 0.032 |
| SWI/SNF pathway abnormality | 1.705 (0.556-5.227) | 0.934 | 0.350 |
| TP53 pathway abnormality | 0.448 (0.145-1.385) | -1.395 | 0.163 |
| PI3K pathway abnormality | 0.834 (0.257-2.704) | -0.303 | 0.762 |
| Cell Cycle pathway abnormality | 2.542 (0.677-9.539) | 1.382 | 0.167 |
| RTK-RAS pathway abnormality | 0.885 (0.285-2.755) | -0.21 | 0.834 |
| TCR signaling pathway abnormality | 2.469 (0.750-8.129) | 1.487 | 0.137 |
| BCR signaling pathway abnormality | 2.322 (0.709-7.609) | 1.391 | 0.164 |
| Cytokine Receptors pathway abnormality | 0.513 (0.131-2.005) | -0.959 | 0.337 |
| Antimicrobials pathway abnormality | 0.354 (0.074-1.684) | -1.305 | 0.192 |
| Natural Killer Cell Cytotoxicity pathway abnormality | 1.664 (0.534-5.188) | 0.877 | 0.380 |

iHR, interaction hazard ratio between treatments and genomic aberrations; TMB, tumor butation burden; ITH, intratumor heterogeneity; CIS, chromosomal instability score; DDR, DNA damage response; TCR, T-cell receptor; BCR, B-cell receptor.
