# Supplementary material for: Exploratory genomic stratification of benefit from first-line immunotherapy-based combination in advanced biliary tract cancer: a biomarker analysis of a randomized phase 2 trial
Source: Front Immunol. 2026 Jul 27;17:1878780. doi: 10.3389/fimmu.2026.1878780 (PMC13454070; doi:10.3389/fimmu.2026.1878780)
Supplement: Supplementary file 3 [file Table3.docx]

Table S3. Predictive values of genomic aberrations for progression-free survival (clinically adjusted).

| Genomic aberrations subgroup | iHR (95%CI) | z-score | P value |
| --- | --- | --- | --- |
| *TP53* mutation | 0.397 (0.121-1.305) | -1.521 | 0.128 |
| *KRAS* mutation | 2.271 (0.573-9.009) | 1.167 | 0.243 |
| *ARID1A* wildtype | 2.765 (0.630-12.125) | 1.348 | 0.178 |
| *PBRM1* muation | 0.443 (0.081-2.412) | -0.941 | 0.347 |
| *TERT* mutation | 0.123 (0.017-0.915) | -2.047 | 0.041 |
| TMB-H | 0.279 (0.085-0.917) | -2.104 | 0.035 |
| CIS-H | 2.736 (0.848-8.832) | 1.683 | 0.092 |
| ITH-H | 1.207 (0.365-3.994) | 0.309 | 0.757 |
| DDR pathway abnormality | 0.300 (0.096-0.943) | -2.060 | 0.039 |
| SWI/SNF pathway abnormality | 1.705 (0.514-5.648) | 0.873 | 0.383 |
| TP53 pathway abnormality | 0.516 (0.157-1.700) | -1.087 | 0.277 |
| PI3K pathway abnormality | 0.607 (0.177-2.078) | -0.795 | 0.426 |
| Cell Cycle pathway abnormality | 2.053 (0.524-8.043) | 1.033 | 0.302 |
| RTK-RAS pathway abnormality | 0.813 (0.238-2.776) | -0.330 | 0.742 |
| TCR signaling pathway abnormality | 2.702 (0.801-9.110) | 1.603 | 0.109 |
| BCR signaling pathway abnormality | 2.651 (0.797-8.822) | 1.589 | 0.112 |
| Cytokine Receptors pathway abnormality | 0.34 (0.080-1.445) | -1.461 | 0.144 |
| Antimicrobials pathway abnormality | 0.373 (0.074-1.889) | -1.191 | 0.234 |
| Natural Killer Cell Cytotoxicity pathway abnormality | 1.944 (0.567-6.660) | 1.058 | 0.290 |

iHR, interaction hazard ratio between treatments and genomic aberrations after adjustment for clinical factors; TMB, tumor butation burden; ITH, intratumor heterogeneity; CIS, chromosomal instability score; DDR, DNA damage response; TCR, T-cell receptor; BCR, B-cell receptor.
