# Supplementary material for: Exploratory genomic stratification of benefit from first-line immunotherapy-based combination in advanced biliary tract cancer: a biomarker analysis of a randomized phase 2 trial
Source: Front Immunol. 2026 Jul 27;17:1878780. doi: 10.3389/fimmu.2026.1878780 (PMC13454070; doi:10.3389/fimmu.2026.1878780)
Supplement: Supplementary file 4 [file Table4.docx]

Table S4. Reproducibility of biomarker selection across 58 n−1 leave-one-out cross-validation folds.

| Candidate feature | Mean interaction P-value | Mean rank | Ranked 1st, n (%) | Ranked Top 3, n (%) | Ranked Top 5, n (%) |
| --- | --- | --- | --- | --- | --- |
| DDR alteration | 0.035 | 1.28 | 45 (77.6) | 57 (98.3) | 58 (100.0) |
| TMB-H | 0.046 | 1.86 | 11 (19.0) | 58 (100.0) | 58 (100.0) |
| *TP53* gene | 0.112 | 3.95 | 0 (0.0) | 31 (53.4) | 51 (87.9) |
| *TERT* gene | 0.126 | 4.88 | 0 (0.0) | 7 (12.1) | 48 (82.8) |
| TCR signaling pathway | 0.144 | 5.36 | 0 (0.0) | 11 (19.0) | 31 (53.4) |
| CIS-H | 0.152 | 6.59 | 1 (1.7) | 4 (6.9) | 20 (34.5) |
| BCR signaling pathway | 0.172 | 8.12 | 0 (0.0) | 0 (0.0) | 3 (5.2) |
| TP53 pathway | 0.171 | 8.26 | 0 (0.0) | 1 (1.7) | 7 (12.1) |
| Cell cycle pathway | 0.174 | 8.43 | 0 (0.0) | 2 (3.4) | 4 (6.9) |
| *KRAS* gene | 0.187 | 8.71 | 0 (0.0) | 0 (0.0) | 5 (8.6) |
| Antimicrobials pathway | 0.201 | 9.38 | 0 (0.0) | 2 (3.4) | 4 (6.9) |
| SWI/SNF pathway | 0.359 | 12.95 | 0 (0.0) | 0 (0.0) | 1 (1.7) |
| Cytokine receptors pathway | 0.345 | 13.14 | 0 (0.0) | 0 (0.0) | 0 (0.0) |
| *ARID1A* gene | 0.365 | 13.19 | 1 (1.7) | 1 (1.7) | 1 (1.7) |
| NK cell cytotoxicity pathway | 0.391 | 14.19 | 0 (0.0) | 0 (0.0) | 0 (0.0) |
| *PBRM1* gene | 0.638 | 16.05 | 0 (0.0) | 0 (0.0) | 0 (0.0) |
| PI3K pathway | 0.764 | 17.47 | 0 (0.0) | 0 (0.0) | 0 (0.0) |
| RTK-RAS pathway | 0.831 | 17.86 | 0 (0.0) | 0 (0.0) | 0 (0.0) |
| ITH-H | 0.873 | 18.33 | 0 (0.0) | 0 (0.0) | 0 (0.0) |

DDR, DNA damage response; TMB-H, high tumor mutational burden; CIS-H, high chromosomal instability score; ITH-H, high intratumor heterogeneity; NK, natural killer.

Note: In each of the 58 folds, one patient was withheld, and all 19 candidate features were re-evaluated on the remaining 57 patients using an unadjusted Cox treatment-by-biomarker interaction model. Features were ranked by interaction P-value (lowest to highest). Bold formatting highlights the two selected biomarkers that robustly ranked at the top across all resampling iterations.
