# Supplementary material for: Exploratory genomic stratification of benefit from first-line immunotherapy-based combination in advanced biliary tract cancer: a biomarker analysis of a randomized phase 2 trial
Source: Front Immunol. 2026 Jul 27;17:1878780. doi: 10.3389/fimmu.2026.1878780 (PMC13454070; doi:10.3389/fimmu.2026.1878780)
Supplement: Supplementary file 5 [file Table5.docx]

Table S5. Overlap and collinearity of the two biomarkers included in the genomic classifier based on DDR alteration and TMB-H.

| Metric | Value |
| --- | --- |
| Total patients | 58 |
| DDR alteration positive | 24 |
| TMB-H positive | 32 |
| Both positive | 16 |
| Both negative | 18 |
| DDR alteration positive / TMB-H negative | 8 |
| DDR alteration negative / TMB-H positive | 16 |
| Overlap among all patients | 27.60% |
| Overlap among DDR alteration-positive patients | 66.70% |
| Overlap among TMB-H-positive patients | 50.00% |
| Phi coefficient | 0.194 |
| Fisher’s exact p value | 0.183 |
| VIF for DDR alteration | 1.193 |
| VIF for TMB-H | 1.586 |

Abbreviations: DDR, DNA damage response; TMB-H, tumor mutational burden-high; VIF, variance inflation factor. Overall overlap was defined as the proportion of tumors positive for both features in the full cohort. Overlap among feature-positive tumors was defined relative to the number of tumors positive for the corresponding feature.
