# Supplementary material for: Exploratory genomic stratification of benefit from first-line immunotherapy-based combination in advanced biliary tract cancer: a biomarker analysis of a randomized phase 2 trial
Source: Front Immunol. 2026 Jul 27;17:1878780. doi: 10.3389/fimmu.2026.1878780 (PMC13454070; doi:10.3389/fimmu.2026.1878780)
Supplement: Supplementary file 6 [file Table6.docx]

Table S6. Baseline clinical characteristics of patients stratified by the genomic classifier.

| Patient chracteristics | SP Subgroup | | | GP Subgroup | | |
| --- | --- | --- | --- | --- | --- | --- |
|  | SAGC (*n* = 22) | GC (*n* = 18) | *P* value | SAGC (*n* = 8) | GC (*n* = 10) | *P* value |
| Age (years), median (Range)  ≥65  <65 | 62 (34, 73)  9 (40.9%)  13 (59.1%) | 64 (32, 74)  9 (50.0%)  9 (50.0%) | 0.935  0.750 | 62 (51, 73)  3 (37.5%)  5 (62.5%) | 54.5 (45, 78)  2 (20.0%)  8 (80.0%) | 0.212  0.608 |
| Sex |  |  | 0.744 |  |  | 1.000 |
| Female | 7 (31.8%) | 7 (38.9%) |  | 3 (37.5%) | 3 (30.0%) |  |
| Male | 15 (68.2%) | 11 (61.1%) |  | 5 (62.5%) | 7 (70.0%) |  |
| ECOG^b^ performance status score |  |  | 1.000 |  |  | 1.000 |
| 0 | 1 (4.5%) | 0 (0.0%) |  | 0 (0.0%) | 0 (0.0%) |  |
| 1 | 21 (95.5%) | 18 (100.0%) |  | 8 (100.0%) | 10 (100.0%) |  |
| Primary tumor type |  |  | 1.000 |  |  | 0.043 |
| Extrahepatic cholangiocarcinoma | 2 (9.1%) | 2 (11.1%) |  | 2 (25.0%) | 1 (10.0%) |  |
| Gallbladder carcinoma | 6 (27.3%) | 4 (22.2%) |  | 3 (37.5%) | 0 (0.0%) |  |
| Intrahepatic cholangiocarcinoma | 14 (63.6%) | 12 (66.7%) |  | 3 (37.5%) | 9 (90.0%) |  |
| Metastases |  |  |  |  |  |  |
| Liver | 15 (68.2%) | 14 (77.8%) | 0.723 | 5 (62.5%) | 7 (70.0%) | 1.000 |
| Lung | 3 (13.6%) | 3 (16.7%) | 1.000 | 2 (25.0%) | 3 (30.0%) | 1.000 |
| Bone | 1 (4.5%) | 2 (11.1%) | 0.579 | 2 (25.0%) | 2 (20.0%) | 1.000 |
| Peritoneum | 4 (18.2%) | 4 (22.2%) | 1.000 | 2 (25.0%) | 2 (20.0%) | 1.000 |
| Distant lymph node | 15 (68.2%) | 8 (44.4%) | 0.200 | 7 (87.5%) | 8 (80.0%) | 1.000 |
