# Supplementary material for: Exploratory genomic stratification of benefit from first-line immunotherapy-based combination in advanced biliary tract cancer: a biomarker analysis of a randomized phase 2 trial
Source: Front Immunol. 2026 Jul 27;17:1878780. doi: 10.3389/fimmu.2026.1878780 (PMC13454070; doi:10.3389/fimmu.2026.1878780)
Supplement: Supplementary file 7 [file Table7.docx]

Table S7. Multivariable Cox regression analyses evaluating the treatment-by-biomarker interaction and the subgroup treatment effect, adjusted for primary tumor type.

| Endpoints | Adjusted Interaction P value^a^ (Overall Cohort, n=58) | Adjusted HR for SAGC vs. GC (95% CI)^b^ (GP Subgroup, n=18) | Adjusted P value (GP Subgroup) |
| --- | --- | --- | --- |
| Progression-free survival | 0.001 | 2.26 (0.65 – 7.89) | 0.202 |
| Overall survival | 0.005 | 3.27 (0.92 – 11.70) | 0.068 |

Note: ^a^ Interaction P values were derived from multivariable Cox proportional hazards models including treatment, biomarker group, their interaction term, and primary tumor type (gallbladder carcinoma vs. non-gallbladder carcinoma) as covariates. ^b^ Hazard ratios (HR) and P values were derived from multivariable Cox models within the GP subgroup, adjusting for primary tumor type.
