# Supplementary material for: Exploratory genomic stratification of benefit from first-line immunotherapy-based combination in advanced biliary tract cancer: a biomarker analysis of a randomized phase 2 trial
Source: Front Immunol. 2026 Jul 27;17:1878780. doi: 10.3389/fimmu.2026.1878780 (PMC13454070; doi:10.3389/fimmu.2026.1878780)
Supplement: Supplementary file 8 [file Table8.docx]

Table S8. Leave-one-out cross-validation summary of the genomic classifier based on DDR alteration and TMB-H.

Part 1. Summary statistics.

| Metric | Value |
| --- | --- |
| Number of patients | 58 |
| LOOCV iterations | 58 |
| Original interaction coefficient | -2.23 |
| Original interaction iHR | 0.11 |
| Original interaction z-score | -3.28 |
| Original interaction p value | 0.001 |
| Group agreement | 58/58 (100%) |
| DDR alteration median z (range; IQR) | –2.09 (–2.72 to 1.86; 0.14) |
| DDR alteration median iHR (range; IQR) | 0.30 (0.19 to 0.34; 0.03) |
| TMB-H median z-score (range; IQR) | –1.98 (–2.71 to 1.58; 0.16) |
| TMB-H median iHR (range; IQR) | 0.32 (0.20 to 0.40; 0.03) |

Part 2. Patient level concordance and fold-specific interaction metrics.

| ID | Original Group | CV Group | Concordant | DDR alteration z-score | DDR alteration iHR | TMB-H z-score | TMB-H iHR |
| --- | --- | --- | --- | --- | --- | --- | --- |
| P01 | SP | SP | Yes | -2.25973 | 0.26784 | -1.9155 | 0.32885 |
| P02 | SP | SP | Yes | -2.29352 | 0.262867 | -2.16371 | 0.285551 |
| P03 | SP | SP | Yes | -2.3926 | 0.247065 | -1.93413 | 0.327156 |
| P04 | SP | SP | Yes | -2.05531 | 0.304004 | -2.16371 | 0.285551 |
| P05 | SP | SP | Yes | -2.06986 | 0.300344 | -1.93195 | 0.327528 |
| P06 | SP | SP | Yes | -2.55591 | 0.227161 | -1.82802 | 0.350748 |
| P07 | SP | SP | Yes | -2.26838 | 0.266976 | -1.91075 | 0.332211 |
| P08 | SP | SP | Yes | -2.09657 | 0.295506 | -1.99545 | 0.314486 |
| P09 | GP | GP | Yes | -2.11107 | 0.294297 | -2.01301 | 0.311022 |
| P10 | SP | SP | Yes | -2.14748 | 0.286037 | -2.03718 | 0.306758 |
| P11 | GP | GP | Yes | -2.05825 | 0.303755 | -1.92022 | 0.32797 |
| P12 | GP | GP | Yes | -2.06549 | 0.302517 | -1.93078 | 0.326061 |
| P13 | SP | SP | Yes | -2.30757 | 0.260866 | -2.16109 | 0.285969 |
| P14 | GP | GP | Yes | -2.06037 | 0.303505 | -1.95429 | 0.321672 |
| P15 | SP | SP | Yes | -1.85882 | 0.343715 | -1.82802 | 0.350748 |
| P16 | SP | SP | Yes | -2.06549 | 0.302517 | -2.10755 | 0.294621 |
| P17 | SP | SP | Yes | -2.09657 | 0.295506 | -1.99545 | 0.314486 |
| P18 | GP | GP | Yes | -2.06114 | 0.303138 | -1.91434 | 0.329022 |
| P19 | SP | SP | Yes | -2.28216 | 0.264541 | -1.9122 | 0.32938 |
| P20 | SP | SP | Yes | -2.09657 | 0.295506 | -2.0474 | 0.304928 |
| P21 | SP | SP | Yes | -2.06986 | 0.300344 | -2.14416 | 0.286658 |
| P22 | SP | SP | Yes | -2.17425 | 0.28164 | -2.08037 | 0.299193 |
| P23 | SP | SP | Yes | -2.1985 | 0.278845 | -1.95061 | 0.323587 |
| P24 | SP | SP | Yes | -2.05825 | 0.303755 | -2.11712 | 0.292995 |
| P25 | GP | GP | Yes | -2.05577 | 0.304008 | -1.9122 | 0.32938 |
| P26 | SP | SP | Yes | -2.06182 | 0.303337 | -2.08502 | 0.298369 |
| P27 | GP | GP | Yes | -2.06549 | 0.302517 | -1.93078 | 0.326061 |
| P28 | SP | SP | Yes | -2.02941 | 0.307834 | -1.91075 | 0.332211 |
| P29 | GP | GP | Yes | -2.06182 | 0.303337 | -1.98402 | 0.316289 |
| P30 | SP | SP | Yes | -1.85882 | 0.343715 | -2.70524 | 0.196449 |
| P31 | SP | SP | Yes | -1.98515 | 0.313512 | -1.92199 | 0.328966 |
| P32 | SP | SP | Yes | -2.0023 | 0.310544 | -2.17424 | 0.280473 |
| P33 | GP | GP | Yes | -2.28151 | 0.266463 | -2.18784 | 0.278321 |
| P34 | SP | SP | Yes | -2.28151 | 0.266463 | -1.91824 | 0.329662 |
| P35 | SP | SP | Yes | -2.06553 | 0.299553 | -1.96719 | 0.320724 |
| P36 | SP | SP | Yes | -2.28626 | 0.265778 | -1.91457 | 0.330342 |
| P37 | SP | SP | Yes | -2.09506 | 0.294502 | -2.10493 | 0.291605 |
| P38 | GP | GP | Yes | -2.07721 | 0.300005 | -1.95562 | 0.318243 |
| P39 | SP | SP | Yes | -2.07721 | 0.300005 | -2.1195 | 0.293232 |
| P40 | GP | GP | Yes | -2.12689 | 0.294086 | -2.00767 | 0.315453 |
| P41 | SP | SP | Yes | -2.22807 | 0.274414 | -1.96098 | 0.321851 |
| P42 | GP | GP | Yes | -2.09807 | 0.296146 | -2.01169 | 0.307881 |
| P43 | SP | SP | Yes | -1.98515 | 0.313512 | -1.92199 | 0.328966 |
| P44 | SP | SP | Yes | -1.99455 | 0.316105 | -2.35531 | 0.252531 |
| P45 | GP | GP | Yes | -2.12689 | 0.294086 | -2.00767 | 0.315453 |
| P46 | SP | SP | Yes | -2.19114 | 0.278174 | -2.04709 | 0.301551 |
| P47 | GP | GP | Yes | -2.05888 | 0.303366 | -1.90987 | 0.327061 |
| P48 | SP | SP | Yes | -2.2359 | 0.270509 | -2.05146 | 0.305243 |
| P49 | SP | SP | Yes | -2.07721 | 0.300005 | -2.10691 | 0.295469 |
| P50 | SP | SP | Yes | -1.96012 | 0.317893 | -1.91457 | 0.330342 |
| P51 | SP | SP | Yes | -2.17861 | 0.282191 | -1.99642 | 0.315311 |
| P52 | GP | GP | Yes | -2.09795 | 0.298788 | -1.96993 | 0.322047 |
| P53 | GP | GP | Yes | -1.87407 | 0.340297 | -1.57609 | 0.403502 |
| P54 | SP | SP | Yes | -2.72079 | 0.192256 | -2.23855 | 0.272788 |
| P55 | SP | SP | Yes | -2.02141 | 0.307196 | -1.957 | 0.322607 |
| P56 | GP | GP | Yes | -2.1337 | 0.289677 | -2.04709 | 0.301551 |
| P57 | GP | GP | Yes | -2.07721 | 0.300005 | -1.94249 | 0.320791 |
| P58 | SP | SP | Yes | -2.21105 | 0.277036 | -1.96845 | 0.320484 |

LOOCV, leave-one-out cross-validation; iHR, interaction hazard ratio; IQR, interquartile range; DDR, DNA damage response alteration; TMB-H, tumor mutational burden-high; SP, SAGC-predominant; GP, GC-predominant; CV, cross validation.
