# Supplementary material for: Exploratory genomic stratification of benefit from first-line immunotherapy-based combination in advanced biliary tract cancer: a biomarker analysis of a randomized phase 2 trial
Source: Front Immunol. 2026 Jul 27;17:1878780. doi: 10.3389/fimmu.2026.1878780 (PMC13454070; doi:10.3389/fimmu.2026.1878780)
Supplement: Supplementary file 9 [file Table9.docx]

Table S9. Bootstrap validation of subgroup-specific treatment effects and treatment-by-subgroup interaction for progression-free survival.

| Metric | Value |
| --- | --- |
| Valid bootstrap iterations | 1000/1000 |
| Median HR in SP subgroup | 0.25 |
| 95% bootstrap interval for SP HR | 0.10-0.50 |
| Proportion of SP HR < 1 | 100.0% |
| Median HR in GP subgroup | 2.92 |
| 95% bootstrap interval for GP HR | 0.86-3.0e+9 |
| Proportion of interaction p < 0.05 | 93.20% |
| Proportion of interaction p < 0.10 | 97.60% |
| Median interaction HR | 0.10 |
| 95% bootstrap interval for interaction HR | 0.02-0.31 |
| Median interaction p value | 0.001 |

Abbreviations: HR, hazard ratio; CI, confidence interval. Bootstrap intervals were defined by the 2.5th and 97.5th percentiles of the bootstrap distribution. Because the bootstrap HR distribution in the GP subgroup was markedly right-skewed, the median rather than the mean is emphasized for this subgroup.
